# Self-management interventions to improve skin care for pressure ulcer prevention in people with spinal cord injuries: a systematic review protocol

**DOI:** 10.1186/s13643-016-0323-4

**Published:** 2016-09-06

**Authors:** Justine Baron, Jillian Swaine, J. Presseau, Arlene Aspinall, Susan Jaglal, Barry White, Dalton Wolfe, Jeremy Grimshaw

**Affiliations:** 1 Clinical Epidemiology Program, Ottawa Hospital Research Institute, Ottawa, ON Canada; 2 Department of Medicine, University of Ottawa, Ottawa, ON Canada; 3 School of Surgery, University of Western Australia, Perth, Australia; 4 Institute for Health Research, University of Notre Dame Australia, Fremantle, Australia; 5 Rick Hansen Institute, Vancouver, BC Canada; 6 Vancouver General Hospital, Vancouver, BC Canada; 7 Department of Physical Therapy, University of Toronto, Toronto, ON Canada; 8 Toronto Rehabilitation Institute, Toronto, ON Canada; 9 Parkwood Institute Research, Lawson Health Research Institute, London, ON Canada; 10 University of Western Ontario, London, ON Canada

**Keywords:** Spinal cord injury, Pressure ulcers, Bed sores, Self-management, Self-care, Skin

## Abstract

**Background:**

Pressure ulcers are a serious, common, lifelong, and costly secondary complication of spinal cord injury (SCI). Community-dwelling people with a SCI can prevent them with appropriate skin care (i.e. pressure relieving activities, skin checks). Adherence to skin care remains suboptimal however, and self-management interventions that focus on improving this have been designed. Little is known on their content, effectiveness, or theoretical basis. The aim of the proposed systematic review is to synthesize the literature on self-management interventions to improve skin care in people with a SCI. Specific objectives are to describe these interventions in relation to their content, effectiveness, theory base, and adherence to reporting guidelines for intervention description.

**Methods:**

The search strategy will combine an electronic search of nine bibliographic databases (MEDLINE, Embase, PsycInfo, CENTRAL, CINAHL, Rehabdata, CIRRIE, PEDro, ERIC) and two trial registers with a manual search of relevant reference lists. Predefined eligibility criteria will be applied in a two-phase selection process involving title and abstract screening, followed by full-text screening. A data extraction spreadsheet will be applied to included papers. Intervention content will be coded using two taxonomies (behaviour change taxonomy; PRISMS self-management support taxonomy). A validated tool (Theory Coding Scheme) and the Template for Intervention Description and Replication (TIDieR) will be used to examine theoretical basis and assess adherence to reporting guidelines for intervention description. A small number of heterogeneous studies are likely to be included in this review therefore a narrative synthesis is planned.

**Discussion:**

This systematic review will help identify the gaps and priorities to guide future research activities in this area.

**Systematic review registration:**

PROSPERO CRD42016033191

**Electronic supplementary material:**

The online version of this article (doi:10.1186/s13643-016-0323-4) contains supplementary material, which is available to authorized users.

## Introduction

A pressure ulcer is an area of skin and tissue damage that is caused by sitting or lying for too long on one part of the body or by the combination of pressure and strain on the skin during certain body movements. They usually occur over a bony prominence [1, 2] and are also referred to as ‘pressure sores’, ‘pressure injuries’, ‘bed sores’, and ‘decubitus ulcers’. They are one of the most common secondary complications for people with a spinal cord injury (SCI) [3, 4]. They occur in 30–85 % of patients during the first month of injury [1], and 85 % of individuals with SCI are likely to experience a pressure ulcer during their lifetime [5]. A large community survey in Canada found that 15 % of people with SCI had experienced two or more pressure ulcers in the last 12 months [6, 7].

The average cost of managing one pressure ulcer in a community-dwelling person with SCI in Ontario has been estimated at CAD $4745 per month [8], with hospital admission costs contributing most significantly (62 %) to these costs. The occurrence of a pressure ulcer often results in a costly cycle of recurrent hospitalizations, surgeries, clinic visits, and home health care needs [9]. They have been found to have a significant effect on physical, social, psychological, and financial aspects of health-related quality of life in people with a SCI [10, 11].

Efforts are underway to improve the prevention and management of pressure ulcers in clinical settings [11–15]. Less attention has been given to the promotion of skin care behaviours in community-dwelling patients with a SCI, despite this being where a large proportion of pressure ulcers develop and worsen. Recommended skin care includes behaviours such as redistributing the loads on the soft tissues (e.g. pressure redistribution/weight shifting/pressure relief activities), preventing skin moisture related to sweat or incontinence, and identifying and reacting to signs of emerging pressure ulcers (e.g. daily skin checks, seeking health care). Other, less skin specific preventive behaviours are also recommended and include dietary intake (nutrition and hydration) and physical activity. Despite efforts to disseminate the importance of skin care [2], research has shown that adherence to these behaviours is suboptimal [16–19], and 29.9 % of pressure ulcers are considered by patients themselves to be associated with shortcomings in their preventive behaviours [17].

Self-management programmes are designed to support people to change their health behaviours [20]. Self-management has been defined as ‘the individual’s ability to manage the symptoms, treatment regimes, physical and psychosocial consequences and lifestyle changes inherent in living with a chronic illness’ [21]. Self-management interventions aim to support patients with three main tasks: (1) the medical management of the condition, (2) coping with the effects of the condition and carrying out usual roles and activities, and (3) managing the emotional impact of the condition [22]. They can be generic or condition-specific, led by a variety of people (e.g. health care professionals, trained volunteers, peers), and delivered to groups or one-to-one. Their content can be variable, and they can be single-component or multifaceted [23–25]. Typically, they consist of more than the provision of education, which alone is often insufficient for behaviour change to occur [26]. Other components in self-management interventions may for example include skills training, action planning, problem solving, self-monitoring, feedback, practical support, training/rehearsal to communicate with health care professionals, and/or social support [27, 28].

Compared to more prevalent chronic conditions with well-established self-management programmes in Canada [29], self-management approaches have only recently begun to gain momentum in the field of SCI. Efforts to survey self-management needs [30] and to understand behaviour change in people with a SCI [31] have begun, and some self-management interventions have been developed [32, 33]. Whilst the focus has primarily been on interventions of an educational nature (i.e. provision of information) [34–36] (e.g. SCI University Online, Spinal Cord Essentials) [37, 38], recent years have seen a growing number of self-management interventions addressing skin care using other strategies. A recent study evaluating a self-management intervention for people with a SCI involved the identification of personally chosen goals, problem solving, motivational interviewing, and provision of information to improve practical knowledge [39]. Another intervention consisted of a risk assessment followed by personalized feedback on level of risk for developing a pressure ulcer [40].

Advances in SCI self-management require certain conditions to be met. First, the use of a common terminology to describe interventions is particularly important for self-management interventions as they vary widely in content [26]. Recent systematic reviews [41, 42] of behavioural interventions have used a behaviour change technique (BCT) taxonomy [43] to describe intervention content according to the ‘active ingredients’ they include. The Practical systematic RevIew of Self-Management Support (PRISMS) taxonomy is another taxonomy published recently that can be used to describe self-management interventions. Not only does the use of a taxonomy to code intervention content facilitate comparisons across studies, but it can also help identify those ingredients associated with greater effectiveness in changing behaviour.

Second, the use of theory in intervention design and evaluation can lead to advances in SCI management. The use of theory is recommended in intervention research [44, 45] as it can help predict the likely pathways of change and therefore the constructs to measure [46]. Some evidence suggests that theory-based interventions may be associated with larger effect sizes [47] although this has not always been confirmed in subsequent research [48]. Previous reviews [49, 50] have used a published checklist (Theory Coding Scheme (TCS)) [51] to assess the theoretical basis of primary studies.

A third requirement for advances in SCI self-management is for past evaluations to include appropriate intervention descriptions. The need for sufficient detail to be reported for replication to be possible has repeatedly been underlined [52–55] and has resulted in the publication of the ‘Template for Intervention Description and Replication’ (TIDieR) reporting guidelines [56]. Without complete descriptions of interventions, researchers cannot effectively use research evidence, nor can health care professionals and patients reliably implement changes that are known to be useful [56].

### Aims

This systematic review aims to review the literature on self-management interventions to improve skin care in people with a SCI and to address the key elements that are important in advancing self-management in SCI. More specifically, we aim to address the following research questions:

1. Which active ingredients are included in self-management interventions targeting skin care in people with a SCI?
2. To what extent are self-management interventions for skin care in people with a SCI theory-based?
3. To what extent do papers presenting the evaluation of self-management interventions for skin care in people with a SCI adhere to the TIDieR reporting guidelines for the description of interventions?
4. How effective are self-management interventions for skin care in people with a SCI and what is the quality of this evidence?

Research question 4 is the most important from a clinician’s perspective. It is listed last as it will be addressed using a more restricted set of study designs than the other research questions (see below).

## Methods

We have followed the Preferred Reporting Items for Systematic review and Meta-Analysis Protocols (PRISMA-P) guidelines [57] in preparing this protocol (see Additional file 1 for further details). Any amendments to this protocol will be described in the final review manuscript with the rationale supporting them. Using a preliminary search of the literature as a starting point, the research team determined a set of eligibility criteria, a list of data sources, and a search strategy to address the research questions above.

### Criteria for including studies in this review

#### Type of studies

Only peer-reviewed studies published in English will be included. Research questions 1 to 3 will be addressed using randomized controlled trials (RCTs) and non-randomized trials with a control group receiving standard care (interrupted time series, quasi-experimental, cohort involving concurrent or historical controls, controlled before and after study) whilst research question 4 on effectiveness will be addressed using the highest quality of evidence, i.e. RCTs only. Unpublished data, abstracts, and conference proceedings will not be included. Studies including no primary evaluation data (e.g. protocols, editorials, systematic reviews) will also be excluded, as well as studies presenting qualitative data only. For studies using mixed methods, only data relating to the quantitative evaluation will be considered.

#### Type of participants

Self-management interventions primarily involving people with a traumatic or non-traumatic SCI will be included. Interventions that involve people with other chronic conditions than SCI will only be included if 50 % or more of the sample is diagnosed with SCI.

#### Type of interventions

Self-management interventions that conform to the definition of self-management proposed by Galdas and coworkers [58] will be included. These authors define self-management interventions as those that are primarily designed to develop the abilities of patients to undertake management of health conditions through education, training, and support to develop patient knowledge, skills or psychological and social resources. As such, only interventions that require patients to be actively engaged, to learn, and/or to develop abilities directly related to skin care for pressure ulcer prevention (e.g. skin checks, weight distribution/shifting) will be included.

Self-management interventions will not be excluded based on the setting in which they are delivered (inpatient and/or outpatient settings, or in the community). Skin care will not need to be the sole focus of the intervention as these are often multifaceted. Studies with a strong primary focus on behaviours less directly related to skin health and more lifestyle-related (e.g. interventions focusing on improving nutritional intake or indicators of physical activity through exercise, smoking cessation) will however be excluded, even though improvements in these areas may indirectly influence skin health. Interventions primarily focusing on pressure ulcer treatment rather than prevention (e.g. supporting patients in the home treatment of existing pressure ulcers) will also be excluded.

#### Type of outcomes

The primary outcome will be a measure of skin care behaviours (e.g. pressure redistribution activities, skin checks), irrespective of the measurement method used. Secondary outcomes of interest include mediators of behaviour change (e.g. beliefs, attitudes, self-efficacy) or self-management-related skills (e.g. problem-solving ability) measured in relation to skin care and pressure ulcer prevention-related outcomes (e.g. prevalence/incidence of pressure ulcers, recurrence of pressure ulcer). Studies including a measure of any of these primary and secondary outcomes will be included in this review.

### Timing

No restriction with regard to the length of follow-up for outcomes will apply.

### Identification of studies

#### Search strategy

Search terms will include terms related to SCI, self-management, and skin care. A comprehensive search strategy has been designed to maximize specificity and sensitivity and in consultation with a librarian (see MEDLINE strategy in Additional file 2). This MEDLINE strategy will be adapted for use in the other large electronic databases (databases 2–5 below). Smaller databases (databases 6–9 below) will be searched using keywords and subject headings if available. The complete search strategy will be peer-reviewed by an independent librarian using an evidence-based checklist [59] and will be made available in the final review manuscript.

#### Electronic search of bibliographic databases

The following bibliographic databases will be searched electronically (no search restrictions will be applied):

1. MEDLINE (In-Process & Other Non-Indexed Citations and Ovid MEDLINE(R), 1946 to present)
2. EMBASE (Ovid, 1974 to present)
3. PsycInfo (Ovid, 1806 to present)
4. Cochrane Central Register of Controlled Trials (CENTRAL)
5. CINAHL (Ebsco)
6. REHABDATA
7. Center for International Rehabilitation Research Information and Exchange (CIRRIE)
8. PEDro
9. ERIC

#### Other data sources

Prospective trial registers (World Health Organization International Clinical Trials Registry; Meta-Register of Controlled Trials) will be searched for relevant studies that may have resulted in publications not identified using the electronic database search detailed above. Related publications will be searched for using the investigator name(s)/project names. Principal investigators will be contacted if necessary.

Relevant unpublished data, abstracts, and conference proceedings will be used to attempt to locate relevant published papers. Reference lists of relevant published protocols, systematic reviews, and of the final list of included studies will be hand-searched.

### Study selection

Search results will be uploaded to the systematic review software Covidence (Alfred Health, Monash University, Melbourne, Australia) [60]. Articles from all searches will be combined and duplicates removed. A two-phase study selection process is planned and involves two reviewers independently screening titles and abstracts first, and then full texts. If the full-text contains insufficient detail for a screening decision to be made, authors of the paper will be contacted for clarification or further information. If the information sought is not provided within 1 month, the paper will be excluded. A PRISMA flow diagram will be used to report the final numbers once the review is complete. A third reviewer will be available to discuss screening outcomes in instances where discrepancies and uncertainties between the two screeners are not resolved.

### Data extraction

Two reviewers will independently extract data from included studies using a pre-determined and piloted data extraction form. Disagreements will be resolved through discussions, with third party adjudication when necessary. Multiple reports of the same study will be combined so that the unit of interest for data extraction and synthesis in this review is the study, not the report.

Standard information on each study will be extracted, as well as data specific to each of the four research questions addressed in this review. Data extraction will include items for the following categories:

- ➢ General information: author names, country of origin, year of publication, journal
- ➢ Study characteristics: study aims, study design (including control groups), eligibility criteria, recruitment and sampling methods, unit of randomization and allocation, blinding
- ➢ Participants: population type, setting, number of participants, baseline characteristics (e.g. age, gender, type of injury, level of injury, comorbidities)
- ➢ Intervention characteristics: duration, timing, frequency, providers, mode of delivery, full description of treatments (including control group) to code treatment content using the BCT [43] and PRISMS [28] taxonomies (research question 1), data on items of the TCS checklist [51] (research question 2) and on the TIDieR checklist [56] (research question 3)
- ➢ Measurements: primary and secondary outcomes of interest measured, time points, unit of measurement, type of measurement, psychometric properties (if applicable), power/sample size calculation
- ➢ Data analysis: type of analyses conducted (e.g. statistical tests, intention to treat versus per protocol)
- ➢ Intervention effects: results of analyses (means, standard deviations, effect sizes, *p* values) (research question 3)

To ensure comprehensive coding of intervention content using the BCT and PRISMS taxonomies, authors of all primary studies will be contacted to obtain copies of existing intervention materials (e.g. detailed protocol, manuals/workbooks, other intervention materials such as presentations made). Additional information or clarifications required for other data extraction items may also be requested on this occasion. To ensure consistency in the interpretation of both taxonomies as applied to the field of SCI, the two coders will independently code a small number of studies from those not selected, prior to coding the included studies.

### Quality assessment

Two reviewers will independently assess risk of bias in RCTs using the tool recommended by the Cochrane Collaboration. This tool focuses on six domains (random sequence generation, allocation concealment, blinding of personnel and outcome assessors, incomplete outcome data, selective reporting, and other sources of bias) and helps estimate selection bias, performance bias, attrition bias, and reporting bias [61]. For each item, studies will be classified as ‘high’, ‘low’, or ‘unclear’ risk of bias. Authors will be contacted for clarifications or supplementary information if necessary. The unclear category will be used for papers for which there is insufficient detail for a conclusion to be reached. Disagreements between the two reviewers will be resolved by discussion. If necessary, a third author will be consulted for arbitration.

As mentioned above (see the ‘Type of studies’ section), non-RCTs will not be considered when examining intervention effectiveness, and their risk of bias will therefore not be assessed.

### Data synthesis

Based on other reviews of self-management interventions [62, 63], we expect diversity in intervention components, outcomes assessed, measurement tools, and time points. With such heterogeneity, computing and pooling effect sizes is unlikely to be meaningful [54]. Instead, tables will be used to descriptively summarize features of the included studies with respect to their design, targeted population, sample size, intervention characteristics, and outcomes measured. Outcomes will be categorized in relation to whether they are mediators of behaviour change (e.g. self-efficacy, beliefs, attitude), skin care behaviours (e.g. pressure redistribution activities), or related to pressure ulcers (e.g. pressure ulcer prevalence, reoccurrence of pressure ulcers). If appropriate, they may also be classified as short term (measured during or within 1 month of the end of the intervention period), medium term (between 1 and 6 months post-intervention), and long-term (6 months or longer post-intervention).

To address research question 1, the type and frequency at which intervention ingredients have been evaluated alone and/or in combination with others will be reported. Data on theoretical basis and adherence to reporting guidelines for intervention description will be provided in separate tables to address research questions 2 and 3. For research question 4, differences in effectiveness will be examined in relation to outcomes, the number and type of intervention ingredients, theoretical basis, and risk of bias assessment results.

## Discussion

No review of self-management interventions for skin care in people with a SCI has been conducted to date, despite the importance of pressure ulcer prevention in this population. Previous reviews have mainly focused on educational interventions [34–36]. In addition to identifying self-management studies that include a focus on skin care and aim to prevent pressure ulcers in people with a SCI, the proposed systematic review aims to describe these interventions with regard to the active ingredients they include and their effectiveness. Describing intervention content with the consistent terminology that the taxonomies used offer will maximize comparability and replicability across studies and will help advance the science of behaviour change in SCI, which in turn can contribute towards improved care and support for people with a SCI. The use of RCTs only to examine effectiveness will help ensure that the conclusions made are tailored to the highest quality of evidence available. The decision to not include qualitative enquiries in this review was based on our preliminary literature search. Very few (if any) were found to have been published so far, and restricting this review to quantitative data was considered unlikely to influence results.

The importance of describing an intervention in sufficient detail to allow for replication has been emphasized in intervention research for a number of years [52–55] and is a necessary condition to advance science. A preliminary review of the literature suggests that some SCI self-management interventions may be poorly described. Poor descriptions of interventions are not uncommon in other fields [64] and may make intervention coding in this review more challenging. This explains the decisions to use one taxonomy containing broader ‘ingredient’ categories [28] than the other [43], and to contact authors to obtain intervention materials.

Examination of the extent to which studies adhere to the TIDieR reporting guidelines for intervention description will allow for further comments and recommendations to be made on the state of reporting in this area of research. Given the TIDieR guidelines [56, 65] are recent, findings from this review will not be used as a reliable indicator of their effect on reporting quality in SCI self-management research. Our focus on them is an effort to underline their importance in building a science of self-management and to gain insights into the intervention details most often reported and neglected so far, as this information may be useful in directing future reporting efforts.

Finally, the examination of the extent to which theory is used in the current SCI self-management interventions will provide the basis for recommendations relating to their design and evaluation. Exploring theory use may provide useful information on the current basis for developing such interventions, as well as the extent to which pathways of change are considered when selecting outcome measurements.

In summary, the proposed review of SCI self-management interventions for pressure ulcer prevention holds the potential to inform practitioners, researchers, and policy-makers by (1) describing variations in practices, (2) reviewing their effectiveness and identifying active ingredients related to larger effect sizes, and (3) identifying gaps and priorities to guide future work in this area. This information is likely to help contribute towards efforts to improve the quality of care and the support that people with SCI receive in the future.

## Additional files

Additional file 1: PRISMA-P (Preferred Reporting Items for Systematic Review and Meta-Analysis Protocols) 2015 checklist: recommended items to address in a systematic review protocol. PRISMA-P checklist populated for this study. (DOC 87 kb) Additional file 2: MELDINE search strategy. List of search terms for the search strategy to run in MEDLINE. (DOCX 14.5 kb)

## Abbreviations

BCT: Behaviour change technique
CENTRAL: Cochrane Central Register of Controlled Trials
CIRRIE: Center for International Rehabilitation Research Information and Exchange
PRISMA-P: Preferred Reporting Items for Systematic Review and Meta-Analysis Protocols
PRISMS: Practical systematic RevIew of Self-Management Support
RCT: Randomized controlled trial
SCI: Spinal cord injury
TCS: Theory Coding Scheme
TIDieR: Template for Intervention Description and Replication
