# Supplementary material for: Self-management interventions to improve skin care for pressure ulcer prevention in people with spinal cord injuries: a systematic review protocol
Source: Syst Rev. 2016 Sep 6;5(1):150. doi: 10.1186/s13643-016-0323-4 (PMC5011862; doi:10.1186/s13643-016-0323-4)
Supplement: Additional file 1: — PRISMA-P (Preferred Reporting Items for Systematic Review and Meta-Analysis Protocols) 2015 checklist: recommended items to address in a systematic review protocol. PRISMA-P checklist populated for this study. (DOC 87 kb) [file 13643_2016_323_MOESM1_ESM.doc]

**Additional File 1.** PRISMA-P (Preferred Reporting Items for Systematic review and Meta-Analysis Protocols) 2015 checklist: recommended items to address in a systematic review protocol*

| Section and topic | Item No | Checklist item |
| --- | --- | --- |
| ADMINISTRATIVE INFORMATION | | |
| Title: |  |  |
| Identification | 1a | The report has been identified as a systematic review protocol in the title |
| Update | 1b | The protocol is not for an update of a previous systematic review, therefore it as not been identified as such |
| Registration | 2 | This systematic review has been registered in PROSPERO; registration number is CRD42016033191 |
| Authors: |  |  |
| Contact | 3a | Authors: Baron, J.S.* ^1,2^, Swaine, J.M.^3,4^, Presseau, J.^1,2^, Aspinall, A.^5,6^, Jaglal, S.B.^7,8^, White, B.^5^, Wolfe, D.L.^9,10^, Grimshaw, J.M.^1,2^  Affiliations: ^1^ Clinical Epidemiology Program, Ottawa Hospital Research Institute, Ottawa, ON, Canada; ^2^ Department of Medicine, University of Ottawa, Ottawa, ON, Canada; ^3^ School of Surgery, University of Western Australia; ^4^ Institute for Health Research, University of Notre Dame Australia;  ^5^ Rick Hansen Institute, Vancouver, Canada; ^6^ Vancouver General Hospital, Vancouver, BC, Canada;  ^7^ Department of Physical Therapy, University of Toronto, Toronto, ON, Canada; ^8^ Toronto Rehabilitation Institute, Toronto, ON, Canada; ^9^ Parkwood Institute Research, Lawson Health Research Institute, London, ON, Canada; ^10^University of Western Ontario, London, Canada.  *Corresponding author |
| Contributions | 3b | Contributions: Justine Baron is the guarantor. All authors contributed towards the design of the systematic review, including the research questions addressed, the development of the eligibility criteria, data extraction items, risk of bias assessment strategy, and plans to use taxonomies and checklists to code intervention content, assess theoretical basis, and evaluate adherence to reporting guidelines. Justine Baron drafted the manuscript, and all authors have read, provided feedback and approved the final manuscript. |
| Amendments | 4 | This systematic review protocol does not represent an amendment of a previously completed or published protocol |
| Support: |  |  |
| Sources | 5a | Financial support**:** The principal investigator on this project, Justine Baron, is funded by the Rick Hansen Institute, a Canadian-based not-for-profit organization committed to accelerating the translation of discoveries and best practices into improved treatments for people with spinal cord injuries |
| Sponsor | 5b | The Rick Hansen Institute funds Justine Baron, the lead on this review. |
| Role of sponsor or funder | 5c | Two protocol authors (Arlene Aspinall, Barry White) are employed by the Rick Hansen Institute and were involved in the design of this protocol, their views are not necessarily those of the Rick Hansen Institute. The conduct of this review is not likely to be affected by the Rick Hansen Institute as this organisation is not currently involved in the funding or design of self-management intervention for skin care. |
| INTRODUCTION | | |
| Rationale | 6 | The prevalence of pressure ulcers in community-dwelling people with a spinal cord injury is high, despite that many can be prevented by appropriate skin care. These pressure ulcers are costly, and can severely reduce the quality of life of people with a spinal cord injury. Self-management interventions have the potential to help people with a spinal cord injury to manage their skin to prevent pressure ulcers from developing. Their design, content and effectiveness have not however been reviewed so far (see manuscript for expanded version) |
| Objectives | 7 | This systematic review aims to review the literature on self-management interventions to improve skin care in people with a SCI and to address the key elements that are important in advancing self-management in SCI. More specifically, the aim is to address the following research questions:  (1) Which active ingredients are included in self-management interventions targeting skin care in people with a SCI?  (2) To what extent are self-management interventions for skin care in people with a SCI theory-based?  (3) To what extent do papers presenting the evaluation of self-management interventions for skin care in people with a SCI adhere to the TIDieR reporting guidelines for the description of interventions?  (4) How effective are self-management interventions for skin care in people with a SCI and what is the quality of this evidence? |
| METHODS | | |
| Eligibility criteria | 8 | Publication status: only peer-reviewed studies. Unpublished data, abstracts and conference proceedings will not be included.  Language: English  Years considered: No time period limitation  Study design: For research questions 1 to 3 we will include randomized controlled trials (RCTs) and non-randomized trials with control group receiving standard care. For research question 4 on effectiveness we will include RCTs only.  Studies with no primary evaluation data (e.g. protocols, editorials, systematic reviews) will be excluded, as well as studies presenting qualitative data only.  Population: Interventions involving 50% or more people with a traumatic or non-traumatic SCI will be included.  Interventions: Interventions that conform to the definition of self-management proposed by Galdas and colleagues will be included. These authors define self-management interventions as those that are primarily designed to develop the abilities of patients to undertake management of health conditions through education, training and support to develop patient knowledge, skills or psychological and social resources. Only interventions that require patients to be actively engaged, to learn, and/or to develop abilities directly related to skin care for pressure ulcer prevention (e.g. skin checks, weight distribution/shifting) will be included. Interventions with a primary focus on behaviours that are less directly related to skin care (e.g. nutrition, physical activity) or on pressure ulcer treatment rather than prevention (e.g. supporting patients in the home treatment of existing pressure ulcers) will be excluded.  Setting: No intervention will be excluded based on the setting in which it is delivered (inpatient and/or outpatient settings, or in the community)  Outcome(s): Primary outcome will be a measure of skin care behaviours. Secondary outcomes of interest include mediators of behaviour change or self-management-related skills measured in relation to skin care, and pressure ulcer prevention-related outcomes. Studies including a measure of any of these primary and secondary outcomes will be included in this review.  Timing: no restriction with regards to length of follow-up after the intervention delivery |
| Information sources | 9 | Nine electronic bibliographic databases: MEDLINE (In-Process & Other Non-Indexed Citations and Ovid MEDLINE(R), 1946 to Present), EMBASE (Ovid, 1974 to Present), PsycInfo (Ovid, 1806 –Present), Cochrane Central Register of Controlled Trials (CENTRAL), CINAHL (Ebsco), REHABDATA, Center for International Rehabilitation Research Information and Exchange (CIRRIE), PEDro, ERIC.  Prospective trial registers:World Health Organization International Clinical Trials Registry; Meta-Register of Controlled Trials (related publications will be searched for/contacted).  Other data sources: Relevant unpublished data, abstracts and conference proceedings will be used to try to locate relevant published papers. Reference lists of relevant published protocols, systematic reviews, and of the final list of included studies will be hand-searched. |
| Search strategy | 10 | Search strategy for Medline provided as an Appendix |
| Study records: |  |  |
| Data management | 11a | It was specified that Covidence (Alfred Health, Monash University, Melbourne, Australia) would be used. |
| Selection process | 11b | It was specified that two independent reviewers would be selecting studies through each phase of the review (that is, screening, eligibility and inclusion in meta-analysis) |
| Data collection process | 11c | Planned methods for extracting data have been included in the report: we have specified that we will use predetermined and piloted data extraction sheets and that two reviewers will independently extract data from included studies. |
| Data items | 12 | We have listed all data items to extract data on (includes general information, study characteristics, participant information, intervention characteristics, outcome measurements, data analysis techniques, results on intervention effects). |
| Outcomes and prioritization | 13 | We will have specified that data will be extracted on behavioural measurements of skin care and pressure ulcer related outcomes. |
| Risk of bias in individual studies | 14 | We have specified that we will use the risk of bias tool made available by the Cochrane Collaboration and we have specified how the data will be used in data synthesis (i.e. for each item, studies will be classified as 'high', 'low' or 'unclear' risk of bias). |
| Data synthesis | 15a | Criteria under which study data will be narratively synthesised relate to risk of bias, key intervention features, outcome type and length of follow-up. |
|  | 15b | No quantitative synthesis is planned |
|  | 15c | No additional analyses are planned |
|  | 15d | Narrative summary is planned |
| Meta-bias(es) | 16 | No planned assessment of meta-bias(es) |
| Confidence in cumulative evidence | 17 | Strength of the body of evidence not assessed |

*From: Shamseer L, Moher D, Clarke M, Ghersi D, Liberati A, Petticrew M, Shekelle P, Stewart L, PRISMA-P Group. Preferred reporting items for systematic review and meta-analysis protocols (PRISMA-P) 2015: elaboration and explanation. BMJ. 2015 Jan 2;349(jan02 1):g7647.*
