# Supplementary material for: Self-management interventions to improve skin care for pressure ulcer prevention in people with spinal cord injuries: a systematic review protocol
Source: Syst Rev. 2016 Sep 6;5(1):150. doi: 10.1186/s13643-016-0323-4 (PMC5011862; doi:10.1186/s13643-016-0323-4)
Supplement: Additional file 2: — MELDINE search strategy. List of search terms for the search strategy to run in MEDLINE. (DOCX 14.5 kb) [file 13643_2016_323_MOESM2_ESM.docx]

**Additional File 2.** Medline search strategy

1. exp spinal cord injuries/

2. spinal cord/

3. cervical cord/

4. exp Spinal Cord Ischemia/

5. exp spinal injuries/

6. exp spinal cord diseases/

7. exp myelitis/

8. paralysis/

9. exp paraplegia/

10. quadriplegia/

11. (central cord syndrome).ti,ab

12. (spinal cord) adj1 (dysfunction? or injur* or disease? or syndrome or trauma or lesion? or lacerat* or transaction? or fracture? or contusion or ischemia).ti,ab

13. (spine or spinal) adj1 (disease? or injur* or fracture?).ti,ab

14. (paralysis).ti,ab

15. (quadr?plegi*).ti,ab

16. (tetraplegi*).ti,ab

17. (paraplegi*).ti,ab

18. (Brown-Sequard).ti,ab

19. (myelopathy).ti,ab

20. (myelitis).ti,ab

21. (SCI).ti,ab

22. or/1-21

23. exp skin ulcer/

24. exp pressure ulcer/

25. ulcer/

26. (bedsore* or bed sore*).ti,ab

27. (skin) adj1 (ulcer* or care or integrity or management or breakdown or wound? or lesion? or promot*).ti,ab

28. (pressure) adj1 (ulcer* or injur* or damag* or sore* or wound*).ti,ab

29. (decubitus or decubital).ti,ab

30. (secondary) adj2 (complication* or condition*).ti,ab

31. (medical complication*).ti,ab

32. or/23-31

33. disease management/

34. primary prevention/

35. secondary prevention/

36. tertiary prevention/

37. preventive health services/

38. Early Medical Intervention/

39. Health promotion/

40. self-administration/

41. self-medication/

42. self-care/

43. risk reduction behavior/

44. exp consumer participation/

45. health behavior/

46. exp patient compliance/

47. self-examination/

48. exp patient-centered care/

49. activities of daily living/

50. exp life style/

51. (self) adj1 (help or manage* or care or determination or administr* or medicat* or treat*).ti,ab

52. (disease management).ti,ab

53. (expert patient*).ab, ti

54. (empower*).ti,ab

55. (promot*) adj4 (health or behavio?r or well-being)

56. (prevent*).ti,ab

57. (participation) adj1 (patient* or consumer*).ti,ab

58. (support).ti,ab

59. (behavio?r*) adj1 (treatment* or therap* or health or modif* or chang* or intervention).ti,ab

60. program evaluation/

61. (program? or programme?).ti,ab

62. (workshop).ti,ab

63. behavior therapy/

64. (training).ti,ab

65. (skill*).ti,ab

66. (change) adj2 (strategies or strategy).ti,ab

67. (patient-cent?red care).ti,ab

68. (patient-focus*).ti,ab

69. (adjustment).ti,ab

70. (activit* of daily living).ti,ab

71. ((life style or lifestyle) adj5 (modif* or chang* or alter* or rehab* or intervention* or management)).ti,ab

72. (education).ti,ab

73. Health education/

74. Consumer Health information/

75. Patient education as topic/

76. (rehabilitation).ti,ab

77. Telerehabilitation/

78. (telemedicine or telehealth or telecare or telerehabilitation).ti,ab

79. (complian*).ti,ab

80. (adheren*).ti,ab

81. or/33-80

82. 32 AND 81

83. exp skin care/

84. or/82-83

85. 22 AND 84
